# Supplementary material for: MIRACUM-Pipe: An Adaptable Pipeline for Next-Generation Sequencing Analysis, Reporting, and Visualization for Clinical Decision Making
Source: Cancers (Basel). 2023 Jul 1;15(13):3456. doi: 10.3390/cancers15133456 (PMC10340358; doi:10.3390/cancers15133456)
Supplement: Supplementary file 1 [file cancers-15-03456-s001.zip › cancers-2350014-supplementary/Supplementary_cancers-2350014_revised.pdf]

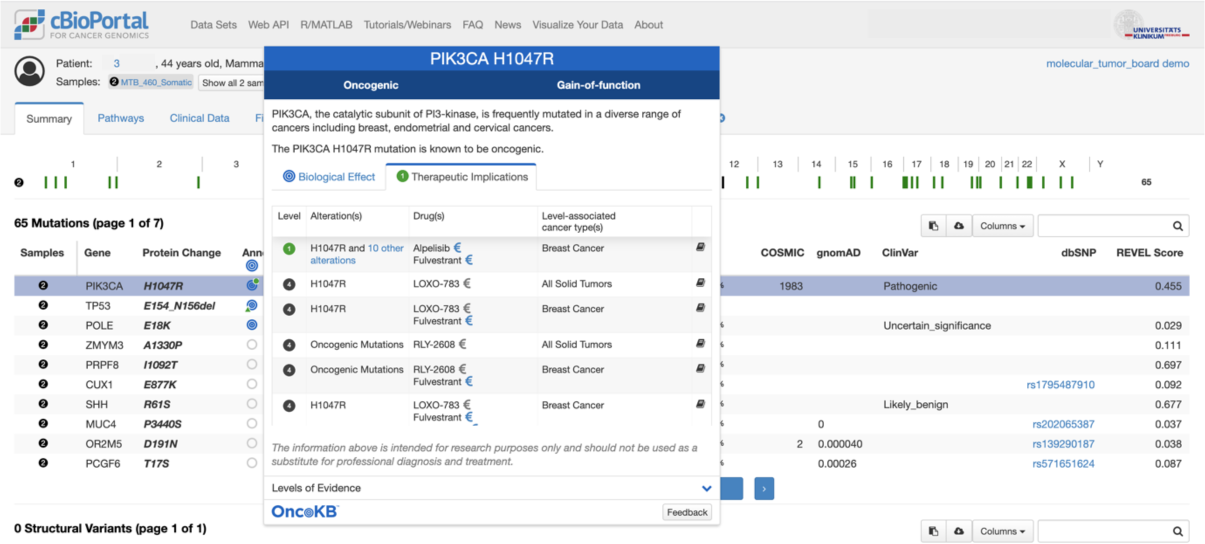

Figure S1: Representation of OncoKB in cBioPortal with the extension of a drug approval status for a specific drug based on the European Medicines Agency (EMA). Drug approval in the European Union is indicated by a € (blue: approved, red: not approved, grey: unknown).

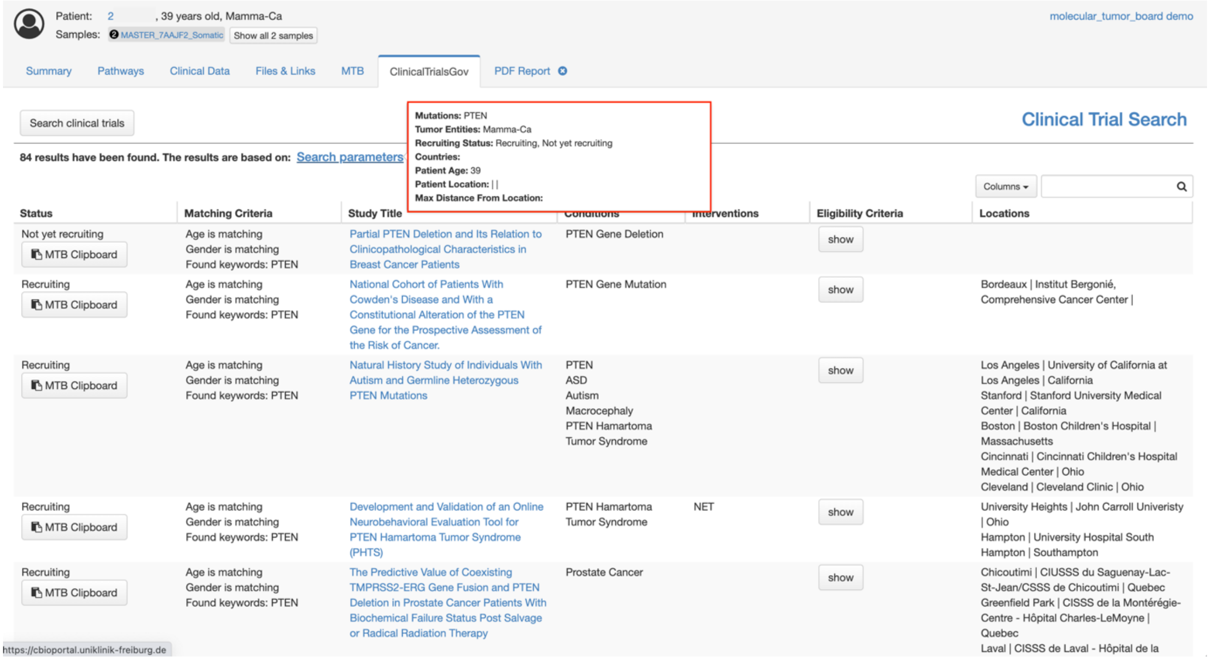

Figure S2: Summary of a specific study query via ClincalTrials.gov integrated within the cBioPortal web interface. The study search criteria, based on genetic and clinical data of a patient are shown in the red labeled box.
